# Supplementary material for: How appraisals of an in-group’s collective history shape collective identity and action: Evidence in relation to African identity
Source: PLoS One. 2024 Sep 6;19(9):e0308727. doi: 10.1371/journal.pone.0308727 (PMC11379368; doi:10.1371/journal.pone.0308727)
Supplement: Attachment — Submitted filename: Response to Reviewers.docx [file pone.0308727.s001.docx]

Many thanks for your positive, encouraging response to the initial version of this paper, and we hope that you agree that acting on them has further improved the manuscript.

The main revisions include (1) the addition of an ethic statement subsection in the Method section across studies on pages 11 and 37 – 38; (2) the addition of extra citations to the Introduction based on Reviewer 1’s suggestions; (3) the correction of the spelling of valence across the manuscript and correction of the labelling of the variable key for collective action (colact) in the correlation table on page 20; (4) the breaking up of longer sentences in the manuscript to improve the readability and clarity; (5) the addition of a paragraph discussing the results of the exploratory factor analysis (EFA) on the appraisals of collective history items on page 32; (6) improving the clarity of the rationale for changing the semantic differential collective history appraisals items used in Study 1 to attitude statements items employed in Studies 2a and 2b on page 36; (7) making the documentary format clearer in the description of the manipulations of Study 1 on pages 13 – 14; (8) the removal of the one-sample t-tests from the manuscript and the inclusion of a link to point readers to the results of the one-sample t-tests in the General Discussion where we draw theoretical implications of the high averages of African in-group engagement on page 61; (9) making clearer (with additional words and sentences) the focus of the exploratory analysis on pages 25 – 31; (10) the addition of words to direct readers to the project OSF site for the results of the pairwise comparisons of the AVOVA effects of historical representation on variables of identification and group-based action on pages 23 – 24; (11) the addition of the course that participants were enrolled in for Study 2b on page 39; (12) the addition of an acknowledgement of the difficulty of trying to replicate the exploratory findings of Study 1 for Studies 2a and 2b on page 56; (13) the correction of the partial eta squared on page 26 to 0.16; (14) the inclusion of a paragraph capturing the moral typecasting model (Gray & Wagner, 2009) in the future research directions in the General discussion on pages 64 – 65; (15) the dropping of the name ‘manipulation check’ for the collective history appraisals items and conceptualising them as dependent variables; (16) the inclusion of a theoretical rationale on page 26 for employing valence as a mediator and subjective importance as a moderator; (17) the addition of sentences in the General Discussion on pages 60 – 61 to suggestions that the appraisals of valence and subjective importance may affect one another (because of the fairly notable correlation between these variables); (18) the addition of a detailed rationale on why we decided to separate the analysis of Studies 2a and 2b (and ultimately present the studies as separate studies) in a paragraph on pages 36 – 37; and (19) the addition of a discussion of the history-as-contrast finding in the General Discussion on pages 59 – 60.

As requested, we have responded to each comment and suggestion of the reviewers in bullet points below each comment/suggestion. **The reviewers’ comments are in italics, and the page numbers we refer to are the page numbers in the revised manuscript with Track Changes (i.e., in the track changes view)**. We hope that this adequately addresses concerns raised regarding the previous version of the manuscript.

**Editor’s Comments**

*Journal Requirements:*

*When submitting your revision, we need you to address these additional requirements.*

*1. Please ensure that your manuscript meets PLOS ONE's style requirements, including those for file naming*

- We have ensured that all PLOS ONE’s style requirements have been met for this resubmission/revision.

*2. Please note that in order to use the direct billing option the corresponding author must be affiliated with the chosen institute. Please either amend your manuscript to change the affiliation or corresponding author, or email us at plosone@plos.org with a request to remove this option.*

- The affiliation has now been changed to the University of Exeter (UoE) which will be billed for this publication as all the research work was conducted at UoE.

*3. Your ethics statement should only appear in the Methods section of your manuscript. If your ethics statement is written in any section besides the Methods, please move it to the Methods section and delete it from any other section. Please ensure that your ethics statement is included in your manuscript, as the ethics statement entered into the online submission form will not be published alongside your manuscript.*

- The ethics statement has now been moved to the method sections, with it now being the first subsection of the method sections (on pages 11 and 37 – 38).

*4. Please review your reference list to ensure that it is complete and correct. If you have cited papers that have been retracted, please include the rationale for doing so in the manuscript text, or remove these references and replace them with relevant current references. Any changes to the reference list should be mentioned in the rebuttal letter that accompanies your revised manuscript. If you need to cite a retracted article, indicate the article’s retracted status in the References list and also include a citation and full reference for the retraction notice.*

- We have now included all citations/references in the reference list.

*Dear Authors,*

*I have received three reviews of the manuscript PONE-D-24-09851 "How appraisals of an in-group's collective history shape collective identity and action: Evidence in relation to African identity". I also read your paper carefully and independently, before looking at the reviews. I want to thank you for submitting your work to PLOS ONE and the three reviewers, each of whom is expert in this area, for their service to the field.*

*As you'll see from reading the reviewers' comments, we all find the document interesting and worthy of praise. At the same time, the comments contain insightful suggestions on how to improve the manuscript. My own reading of the work leads me to agree with this general assessment. That's why I'd like to suggest that you revise the document before it is finally accepted. It is particularly important to include in your cover letter a detailed explanation of your decisions regarding how you have dealt with these comments.*

- Many thanks for your positive, encouraging response to the initial version of this paper, and we hope that you agree that acting on them has further improved the manuscript. As requested, we have responded to each comment and suggestion of the reviewers in bullet points below each comment/suggestion. The reviewers’ comments are in italics, and the page numbers we refer to are the page numbers in the revised manuscript with Track Changes (i.e., in the track changes view). We hope that this adequately addresses concerns raised regarding the previous version of the manuscript.

**Reviewers’ Comments**

**Reviewer 1**

*It is a very interesting work, with data from a cultural region where there are few studies and from one of the great countries of Africa.*

- We thank you for the positive assessments of our work.

*They should review and integrate more articles on social and national identity and collective memory, not only focuse on weird nations,for example those of Rosa Cabecinhas (author cited above) on Lusophone Africa. see also10.18800/psico.201001.005 and see this monograph doi 10.5964/jspp.v5i2.895 and thios one doi.org/10.1111/ajsp.12376*

*Also the recent ones by JH Liu and colleagues on collective memory.*

*It is central for a tru open science perespective to review not only mainstream anglophone authorsless i have forwarded to articles on africa, asia and latin America*

- Thank you for your suggestions which have now been included in the citations in the introduction, and add to the wide range of non-WEIRD research already cited, such as Makanju et al., 2020, 2023; Licata et al.,2018 (of which Cabecinhas and Liu JH were co-authors); Cabecinhas et al., 2011; Klein & Licata, 2003; Bulhan, 1985b, 2015; Rodney, 2012; Fanon, 1967; Figueiredo et al., 2018; Khan, Svensson, Jogdand & Liu, 2017; Liu et al., 1999; etc.
- More precisely, we have added (1) the citations of Figueiredo et al., 2017 and Ionescu et al., 2021 (citations 11 and 12) to the ‘Historical representations and social identities’ subsection of the Introduction; and (2) the citations of Rottenbacher & Espinosa, 2010 and Roth et al., 2017 (citations 23 & 24) to the first paragraph of the ‘Appraising the in-group’s collective history’ subsection of the Introduction.

*Manipulation has limited effects and the moderate mediation is not replicatedThe transparency of the authors should be reinforced in my opinion and the article should be published*

- Thank you for your positive assessment of our manuscript.

*The hypothetical deductive model is a myth and I suggest that the authors better explore their data.*

- It was a little unclear to us whether the reviewer was referring to the model in the paper or the more general hypothetico-deductive paradigm of research. In either case, the paper does include a combination of confirmatory and exploratory analyses, including much more transparent exploration than is typically reported in experimental studies.

*First, since African social identity does not change, it can be used as a dispositional variable.*

*Previous studies have shown that high social identity leads to minimizing or rejecting negative information - for a relationship between collective culture and national identity see Klein.*

*Looking for previous studies and relying on them I would contract this effect - I suggest using tertiles because Klein found a curvilinear effect. see doi 10.1111/j.2044-8309.2011.02028.x*

- Indeed, in our exploration of our data, we have treated African identity as a dispositional variable and we have consistently found that it does not moderate any effects. Ultimately, our data is openly available on the project OSF site, so the reviewer is welcome to explore them in ways not covered in the paper.

*Second, I suggest to do a meta-analytic integration of the three studies and provide an estimate of the relationship between identity and valence and the other variables. You can use fixed model and this can be accomplished with excel no nedd of CMA o R*

- We appreciate this suggestion. However, we do feel that the current analysis fits the purpose of understanding the multi-dimensional nature of collective history appraisals and their relationship with in-group engagement. More importantly, meta-analytic syntheses across original studies are most useful when (1) there are non-zero effects, and (2) there is some variation in those effects, leaving some ambiguity about overall inferences. In the present case, the inference is clear: the manipulations do not have reliable direct effects on any of the ingroup engagement outcomes. A meta-analyses of these hypothesised effects would therefore almost certainly reconfirm this point, and thus be redundant. Therefore, we will not be doing any additional analysis. Ultimately, our data is available on the Project OSF site and we hope other individuals can engage with them in different and useful ways.

*Third, use and aggregate the African collective esteem scale and see if it is identity and collective esteem that predict collective action using multiple regression or SEM.*

*Of course also you can explore what is specifically related to a positive valence of african past (collective identity or esteem) see 10.1017/S1138741600002456*

- We thank you for this suggestion. However, we did not follow the specific rationale for this analysis, and are hesitant to add unfocused exploratory analyses to the already lengthy paper. If the reviewer is curious about specific analyses that we do not currently report, our data are available on the Project OSF site and we hope other individuals can engage with them in different and useful ways.

*Fourth, make the scale of collective action more explicit, what is the we (Nigeria, Africa...) what are the claims and what is the enemy of this action.*

- We have copies of the questionnaires of the studies on the project OSF site at OSF | How appraisals of an in-group's history shape collective identity and action: Evidence in relation to African identity. Our studies were on the African identity (and not the Nigerian identity) and all the collective action items had clear and explicit targets. For example, the items on consciousness-raising were directed at making other Africans aware of African history. As another example, the items on social competition were directed at Africans competing with the West. Additionally, the items on collective action are explained adequately in the methodology sections of the studies.

*Minor points are said valance instead of valence*

*A table is written colact 9 and it is 8*

*Figures have explanatory paragraphs in bold type and this is a mistake*

- Thank you for pointing these out. We have now corrected these.

**Reviewer 2**

*I have now reviewed the manuscript entitled “How appraisals of an in-group’s collective history shape collective identity and action: Evidence in relation to Africa” and my general thought is that it is an interesting, well-written and well-analysed paper. I do have some minor comments and suggestions, however nothing substantial.*

- We thank you for the positive feedback on our manuscript and the outcome of the review.

*Here are some general comments, mostly in order of appearance.*

*1. Throughout the text, there are some hard-to-read sentences, often because they are too long. This is already the case with the very first sentence, especially the part “how group members engage with their in-groups in terms of how much they identify with the in-group”.*

- Thank you for this comment. We have reviewed the manuscript to break up longer sentences to improve the readability and clarity (including the sentence mentioned).

*2. On p. 6, the authors propose a multidimensional approach based on thirteen dimensions, themselves clustered under four superordinate dimensions. This seems like an important conceptual framework that then disappears from the discussion. A discussion about what their factor analysis reveals in relation to their conceptual framework would be worth having. For instance, why did the richness dimension end up loading (after rotation) on the importance factor? Also, I was wondering why they did not use the same items for Study 2A and 2B?*

- We have now included a paragraph discussing the results of the exploratory factor analysis (EFA) on the appraisals of collective history items on page 32. Specifically, we discuss what the results means with the richness (consisting of depth, temporalty and existence) superordinate dimension loading unto the subjective importance dimension.
- This present research was part of a body of research of the first author’s doctoral thesis. Therefore, at the start of Studies 2a and 2b, a separate quantitative study had been completed on the scale confirmation of appraisals of collective history items (employing a confirmatory factor analysis - CFA). Consequently, we thought it made sense to use the confirmed scale items for subsequent work on the thesis, especially as the factor structure (of the CFA) matched that of the EFA results in Study 1. Moreover, the confirmed scale items were attitude statement items. Hence, we reasoned that in most social psychological research the attitude statement format is used instead of the semantic differential format (employed in Study 1), so we thought this was an additional advantage in using the confirmed scale items – to follow the norm of most studies in social psychological research. We have now made this rationale clearer by adding additional words on page 36 where we introduce the changes of measures to the appraisals of collective history. This is in addition to what is on page 41 under the materials section in the ‘Collective history appraisals’ subsection which also points out this change.

*3. On p. 13, the format of the material is not clear. Even though the authors talk about “documentaries”, it could be either written, audio or video documentaries. I would suggest the authors describe the format already in the introduction.*

- We have decided to make the documentary format clearer by adding the word ‘video’ in key places in the description of the manipulation/treatments on pages 13 and 14.

*4. In experiment 1, the control condition ends up being a not very good “control”, as it receives the highest scores on all collective historical appraisal dimensions. But more importantly, it is also not different from some of the precolonial history conditions on certain levels (for example it is as positive as the positive precolonial history). Once these manipulation checks revealed this issue, it might have been better to remove it from the analyses and focus on the comparison of interest, which is the two types of precolonial history and their influence on identification and group-based action. Indeed, an ANOVA including the three conditions might not reveal underlying differences between the two historical conditions on the count of this third “in-between” control condition.*

- We thank you for this comment. Indeed, the goal of introducing a control was to rule out positivity as the factor that impacted in-group engagement. Hence, we operationalised the control to be as positive as the positive history condition. We explained this on pages 13 – 14 of the method section under the 'Materials’ subsection. Furthermore, we believe it is more transparent scientifically to leave the control condition in the analysis for two reasons. First, our exploratory analysis that unveiled the history-as-inspiration and history-as-contrast results showed that the control condition was not relevant in comparison to the positive history condition in relation to increases or decreases in in-group engagement. Second, we feel it is good for other researchers to know what did not work for the advancement of future research. We feel this is very important in such novel investigations in a hard-to-reach population for the progress of science. Altogether, we feel it is best to include the control condition as part of our methodology and statistical analysis in Study 1.

*5. On p. 23 and 49, the authors compare participants’ scores to the midpoint of their scales. To be honest I do not understand the rationale and do not believe it to be relevant. Is there a theoretical (or an empirical) assumption that the population would be centred on the mean point? If yes, this needs to be discussed before. But in many countries, I would not expect measures of identity to be centred around the midpoint. I would prefer a section on descriptive data at the beginning of each result section describing the scores.*

- We included these analyses in the result sections in order to be able to discuss the high averages as a finding in the General Discussion as a theoretical implication of our findings. This was because of the commonly held view that Africans negatively evaluate African identity and are not willing to take progressive action on behalf of the African group or collective (i.e., a collective action problem). Please see this discussion on page 61 in the General Discussion. In other words, to statistically determine the relevance of African identity to our sample, we decided to conduct one-sample t-tests to examine if scores were significantly higher than the mid-point of in-group engagement scales. Moreover, the scale's midpoint is meaningful because it is a bi-polar response scale, so the midpoint represents neutrality, and significantly higher scores represent qualitatively positive attitudes.
- Altogether, as a compromise, we have decided to remove the results of the one-sample t-tests from the manuscript and point readers to these findings in the supplementary materials when we discuss the theoretical implications of the high averages for the variables of identification and group-based action in the General Discussion. Please see page 61 for this change and the Project OSF site (OSF | How appraisals of an in-group's history shape collective identity and action: Evidence in relation to African identity) for the new supplementary material titled ‘Results of one-sample t-tests on identification and group-based action variables for Studies 1, 2a & 2b’.

*6. On p. 25 (and across study 1 in general), the authors seem to consider their manipulation as a manipulation of the appraisal of subjective importance. However, they proposed similar documentaries, with simply a change in valence. It is not clear to me how this change in valence should also be considered a manipulation of the perceived importance.*

- We think this is a misunderstanding of how we explored our data in Study 1. Therefore, we have now made it clearer (in the section of the manuscript that you refer to) that the focus of our exploratory analysis is on the measured – and not the manipulated – appraisals of collective history, especially the measure of the appraised valence and subjective importance. These changes can be found in the results section under the ‘Exploratory analysis: the role of the measured appraisals of collective history in the relationship between historical representations and identification/group-based action’ subsection on pages 25 – 31. More importantly, in the original manuscript, we made it clear in the Discussion section of Study 1 (please see page 31 – 32) that we considered the manipulation of historical representations as primarily a manipulation of valence. We have now also included this in a sentence on page 26 to make this clear in the results section where we talk about our exploratory approach.
- Additionally, we need to note that in the first iteration of this manuscript (within the co-authors) and even the first author’s research talks, for example, these conditions were named prestigious precolonial Africa and narratives of precolonial wrongs of Africans. However, we changed the names to positive and negative precolonial history as we felt that would make our operationalisation much simpler to understand. This change points to the ease of categorising collective history into valence (which is in line with previous research). Nonetheless, at the commencement of this investigation (conceptually and before data collection), we were interested in observing which of the dimensions (i.e., valence, subjective importance, richness and clarity) of collective history were going to be impacted the most by the manipulation of historical representations employed in Study 1. In other words, we did not have a priori expectations as to which dimensions would be varied by the manipulations employed and so we explored this in Study 1.

*7. On p. 31, I would have like the authors to reflect on why the positive history manipulation led to a smaller rating of importance than the negative history manipulation. Furthermore, in the paragraph about the “control” condition (which is not really a “control”), I would have liked to see a discussion about how these results affected their further analyses (not necessary if the authors take on board my suggestion on point 4 and remove the condition for their analyses).*

- From page 25, we switched our focus to the exploratory analysis of the role of the measure appraisals of collective history in the relationship between the manipulation of historical representations and identification/group-based action. This analysis produced the findings of history-as-contrast and history-as-inspiration which then formed the focus of our discussion on pages 33 – 34. As to why the positive history condition led to lower ratings of subjective importance compared to the negative history condition, we point to the analysis presented on pages 20 and 22. Specifically, we conducted pairwise comparisons of the ANOVA effects of historical representations on appraisals of collective history in Study 1. As you will see in Table 3 there was no significant difference for participants in the positive and negative precolonial history conditions on the appraised subjective importance of African history. This non-significant difference was why we did not take it as a key finding and was why we did not discuss it. The only pairwise comparison that had a significant difference between the positive and negative precolonial history conditions was the effect of historical representation on appraised valence (with the positive history condition having a higher score; please see Table 3). Additionally, we have now included all of the pairwise comparisons of ANOVA effects of historical representations on variables of identification and group-based action in the supplementary materials in the document title ‘Study 1_Pairwise comparisons of ANOVA effects of historical representation on variables of identification and group-based action ’ on the project OSF site at OSF | How appraisals of an in-group's history shape collective identity and action: Evidence in relation to African identity . We direct readers to these results via a link to the project OSF site on pages 23 – 24. Altogether, these findings revealed unanimous null effects when comparing participants in the positive and negative precolonial history conditions.
- We have explained the rationale behind our control condition and why we prefer to retain it in the manuscript in our response to point 4.

*8. For studies 2b, students were enrolled in which course? Psychology?*

- We have now included that the students in Study 2b studied psychology.

*9. On p. 33, the authors say that they changed their measures of appraisals from semantic differential to attitude statements, but they do not say why. It makes comparisons between studies difficult.*

- We have addressed this point in our response to point 2 and have also made it clear in the manuscript why we adopted the attitude statement format. Please see the second half of our response to point 2 (by Reviewer 2). We have also now acknowledged this point (of comparisons between Study 1 and Studies 2a and 2b being more difficult) on page 56 where we tried to replicate the findings of Study 1 (i.e., history-as-contrast and history-as-inspiration) for Studies 2a and 2b.

*10. On p. 52, the authors propose that one possible reason for the null effects may be the relative weakness of the manipulation of the subjective importance of African history. However, in study 1, there was no manipulation of the importance, and yet they found it moderated the effect of valence. Furthermore, the manipulation did work in both studies 2a and 2b. In other words, no manipulation (study 1) revealed an effect and manipulation (studies 2) revealed no effect. I think this needs to be discussed a bit further.*

- You are referring here to the discussion section of findings for Studies 2a and 2b. This is not the General Discussion for all studies. Therefore, to improve the clarity of the paragraph on page 57, we have included ‘Studies 2a and 2b’ in the sentence (… the null effects in Studies 2a and 2b…). Moreover, in Study 1, the findings of history-as-inspiration and history-as-contrast were from the measured or appraised subjective importance of African history (and not from any manipulation as there was no manipulation of subjective importance in Study 1). Additionally, we have clarified this in our response to point 6, and have now also added some extra words on page 58 when we refer to the moderated mediation findings in Study 1 in relation to the findings of Studies 2a and 2b. These additions hopefully clarify that we refer to the measured appraisals of subjective importance in Study 1.

*11. At the beginning of the general discussion on p. 53, the authors indicate finding null effects in experiment 1. I would have liked some reflection as to why this was the case (or at least to do it in the discussion of that study).*

- This portion of the General Discussion talks about the null effects of historical representations on identification and group-based action without considering the role of the multi-dimensional nature of collective history appraisals. This was why we did not focus on discussing these null effects. Rather, we focused on exploring the role of collective history appraisals – which is the primary focus of this present research – in the relationship between historical representations and identification and group-based action. This exploration revealed the findings of history-as-contrast and history-as-inspiration which we then discussed in detail in the discussion of Study 1. To make this approach clearer in the revised manuscript, we have added extra words/sentences on pages 25, 33 and 59 to underline our focus on the multi-dimensional nature of collective history appraisals in the experiment in Study 1.
- Nonetheless, looking at the studies holistically, in the future research directions subsection of the General Discussion we proposed a longitudinal research design which should increase the strength of the manipulations of historical representations (and may not lead to null effects) as participants will have a more prolonged, repeated exposure to the treatment (instead of the 5-minutes videos employed in the present research). We also point out that it would be good for future research to utilise real-life social processes that influence in-group members’ appraisals of collective history, such as investigating the role of group discussions. These points can be found on pages 64 – 65.

*Minor details.*

*- p. 5 valance should be “valence”*

*- p. 25, first line. The partial eta square should be .016, not .015.*

*- p. 26-27. The parenthesis “(and not the African Savannah condition)” should be placed earlier in the sentence as it reads as if it the comparison between negative history and Savannah and not positive history and Savannah (there is a similar problem on p. 28).*

- We have now changed throughout the manuscript valance to valence
- We have corrected the partial eta squared on page 26 to 0.16
- We have now removed the parenthesis altogether to improve the clarity

**Reviewer 3**

This paper aims at investigating the impact of African history appraisals on Nigerian participants’ social identification and group-based action. It presents three experiments in which versions of Africa’s past were manipulated. One of the strong points of this paper is the use of – rather large – Nigerian samples, given the scarcity of research with African participants. In addition, the research question is original and, in my view, highly relevant. The theoretical intro is appropriate, too. The confirmation (based on a former qualitative study) of a typology of appraisals of history, alone, is an important contribution. Results do not meet all the expectations as studies 2 and 3 failed to replicate the interesting pattern found in Study 1, but these results are presented and discussed in a very honest and transparent manner. Globally, I believe this paper deserves to be published. However, I'd like to highlight a few critical points which, once considered, could make it even better.

- We thank you for the positive and encouraging feedback on our manuscript and the outcome of the review.

*- A question that remains open throughout the paper is what exactly was manipulated through these excerpts of videos. They are presented as either positive, negative, or neutral. But the nature of “positive” or negative” should be better explained, in my view. Positive could refer to a dimension of morality, but also of agency, competence, etc. If we refer to Gray & Wegner’s moral typecasting model (2009), it seems that Africa is depicted as agentic and moral in the positive video, whereas it is depicted as a negative moral agent in the negative one? The link with social identification seems relevant since people are probably reluctant to identify with an immoral ingroup, but perhaps less so for collective mobilization because the difference in agency between these two experimental conditions is far from obvious. A comparison between a positive agent and negative patient (i.e. victim) moral typecasts would have made sense. I think the paper would benefit from some more explanations about this, and from a deeper discussion.*

- Looking at the peculiarities of the African context and the unresolved legacy of colonialism, we set out to vary historical representations of precolonial Africa (especially to expose participants to narratives of prestigious colonial Africa) to give a lie to the ubiquitous colonialist narrative of Africa (which is dominant in history curricula across Africa). This approach was hopefully clear in the introduction section.
- Moreover, the findings from Study 1 showed that the manipulations of historical representations varied the superordinate dimension of valence which consisted of the dimensions of positivity, pleasantness and glory. Therefore, we acknowledged on pages 26 and 32 that the manipulations primarily varied valence.
- Furthermore, we are aware of Gray & Wagner’s moral typecasting model (2009) and some of the first author’s ongoing work with colleagues in Europe is already working on this model in relation to history and the African context. However, we cannot say that the manipulations of historical representations clearly varied morality especially when looking at the content of the positive precolonial history. Altogether, we do not have a clean variation of (im)morality (and by extension, agency or patiency and competence or incompetence) in the manipulations of historical representation in our study. More importantly, in our study, the moral typecasting model does not explain the findings of history-as-contrast and history-as-inspiration in relation to the measured or appraised valence of African history. The key determining explanation is the role of the measured or appraised subjective importance of African history. This is a unique contribution especially its corroboration with the qualitative findings in Makanju et al., 2023.
- Nonetheless, since this point poses a very interesting empirical question and is one of ongoing research for the first author, we have decided to include a paragraph capturing the moral typecasting model in the future research directions in the General discussion on pages 64 – 65 (the third point on the future research).

*- I think it is inappropriate to present measures of historical appraisal as manipulation checks. On the one hand, they seem too remote from the actual manipulations to be presented as such. And if they are used to check whether a manipulation was effective or not, there should be a conclusion about this effectiveness, which is missing. On the other hand, manipulation control measures cannot be used to test hypotheses. Thus, in this paper, valence cannot be presented as a manipulation check and then used as a mediator.*

- We have decided to drop the name manipulation check across our studies. Conceptually, across studies, we now call the (1) independent variable of the video documentaries historical representations, and (2) collective history appraisals dependent variables (of specific appraisals of African history).

*- 25. The decision to use subjective importance as a moderator is justified on the basis that there was no strong effect of the manipulation on this variable. I think a theoretical explanation is also needed.*

- The reasoning was based on (1) simultaneous/ongoing qualitative research that found history being used as a contrast and inspiration at the time of our research, and (2) speculation that the influence of our valence appraisal of anything will be greater if we also appraise that thing as important to us, which is similar to appraisal theories of emotion and attitude theories. We have now included this second rationale on page 26 in the section that explains the exploratory analysis. We still leave the first rationale on page 34 in the discussion of the findings of Study 1 since it was simultaneous/ongoing research at the time of this research.

*- 26. As I understand, only one contrast was entered into the analysis (comparing the positive to the other two conditions). I’m not an expert in contrast analyses but I think one should always enter all the contrasts (two contrasts for 3 conditions), either polynomial or Elmert. And it would make more sense to compare the two experimental conditions (linear contrast) and the two experimental conditions to the control condition (quadratic contrast). If there is a justification to do otherwise, it should be better explained.*

- We think this is a misunderstanding of our analysis approach as there were two contrasts entered per analysis with dummy coding (through the PROCESS macro extension on SPSS). Specifically, in each analysis, the negative precolonial condition was compared to the positive precolonial condition (the first contrast) and the African Savannah condition was compared to the positive precolonial condition (the second contrast).

*- The positive correlation between subjective importance and valence should be considered in the interpretations. People who hold negative representations of African history may lower its importance.*

- Thank you for this suggestion which has now been included in the General Discussion on pages 60 – 61 (at the end of the penultimate paragraph).

*- I don’t understand clearly why Studies 2a and 2b were presented as distinct studies since it seems to be the same design, same material used for manipulation, same measures, same kind of population (Nigerian adults), etc. Combining the two samples would increase statistical power and simplify the presentation of results. Alternatively, this choice could be better justified.*

- We have now given a detailed rationale for why we decided to separate the analysis of Studies 2a and 2b in a paragraph on pages 36 – 37. Essentially, since we could not establish uniformity in the procedures of Studies 2a and 2b, we decided to analyse these studies separately.

*- In Discussion, I think the important finding that participants who thought African history was not important seemed to use it to contrast their identity with it deserves some more explanation (it seems less intuitively obvious than the assimilation effect found in the positive condition when history was considered important).*

- We discussed this finding in the discussion of Study 1 on page 33 – 34. Given that the exploratory findings were not replicated in Studies 2a and 2b, we thought it should not be central to the General Discussion. Nonetheless, we now revisit this (with an additional point/sentence on modernist or futurist identities) in the General Discussion on pages 59 – 60.

*- 55. I found the discussion of non-expected results very honest, cautious and nuanced.*

*In short, I really enjoyed reading this article and found many aspects to like in it. However, there is some room for improvement. I hope my comments will help the authors in this process. I wish them all the best in pursuing this great line of research!*

- We thank you for your positive assessments of our research and useful feedback and suggestions. We have tried our best to implement most of them and hope the manuscript is in better shape now.
